# Patients’ satisfaction with outpatient pharmacy services and associated factors in Debre Tabor comprehensive specialized hospital, Northwest Ethiopia: A cross-sectional study

**DOI:** 10.1371/journal.pone.0262300

**Published:** 2022-01-05

**Authors:** Mulugeta Molla, Woretaw Sisay, Yared Andargie, Belayneh Kefale, Pradeep Singh

**Affiliations:** 1 Pharmacology and Toxicology Unit, Department of Pharmacy, College of Health Sciences, Debre Tabor University, Debre Tabor, Ethiopia; 2 Clinical Pharmacy Unit, Department of Pharmacy, College of Medical and Health Sciences, Bahir Dar University, Bahir Dar, Ethiopia; 3 Pharmaceutical Chemistry Unit, Department of Pharmacy, College of Health Sciences, Debre Tabor University, Debre Tabor, Ethiopia; University of South Australia, AUSTRALIA

## Abstract

**Introduction:**

By measuring patients’ satisfaction, providers can gain insight into several elements of health care services, including the effectiveness of their care and the level of empathy they exhibit. The aim of this study was to assess patient satisfaction with pharmaceutical services and associated factors in public hospitals located in Northwestern Ethiopia.

**Methods:**

An institution-based quantitative cross-sectional study was used. The study was carried out in an outpatient pharmacy from January 1–June 30, 2021. Participants were selected by a systematic sampling technique. The IBM SPSS statistical package (version 23) was used to enter and analyze the collected data. The findings were presented using descriptive statistical methods. To find factors linked to satisfaction, binary logistic regression was used.

**Results:**

The final analysis included a total of 401 samples. More than half of the participants (229, or 55.1%) were female. The overall mean score of satisfaction was 30.6 out of a maximum of 100 scores. By taking this mean score as a cut-off point, 204 (50.9%) of the study participants had satisfaction with the outpatient pharmacists’ service. Participants’ responses scored on the uncomfortable and inconvenient waiting areas [AOR = 0.31; 95%CI, (0.13, 0.49)] were found to be negatively associated with the level of patients’ satisfaction. Also, the unavailability of medications [AOR = 0.12; 95%CI, (0.02, 0.37)] was negatively associated with the respondent satisfaction. Uncomfortable and inconvenient private counseling areas [AOR = 1.37; 95%CI, (0.79, 4.42)] showed a negative association with their satisfaction.

**Conclusion:**

Patients’ satisfaction levels with pharmacy service were found to be greater than 50%. The socio-demographic characteristics of patients have no association with their level of satisfaction, but their perception of uncomfortable private counseling areas and waiting areas was negatively associated with their satisfaction.

## Introduction

Patient satisfaction and the patient experience of care are top priorities for healthcare organizations. After all, an important quality domain used to measure hospital performance is patient experience of care. By measuring patients’ satisfaction, providers can gain insight into several elements of health care services, including the effectiveness of their care and the level of empathy they exhibit. Patients’ satisfaction is the level of contentment that customers have after using a service, according to certain scholars, despite the fact that there is no consensus among litterateurs on how to describe the idea of patients’ satisfaction in healthcare [1]. Patients’ satisfaction is defined as a patient-reported outcome measure in Avedis Donabedian’s quality assessment paradigm, whereas patient-reported experiences can be used to assess the structures and processes of treatment [2]. Analyzing patients’ satisfaction assists health-care managers in determining the quality of care provided and identifying areas for improvement [3].

Through the implementation of hospital reform guidelines, the Ethiopian Federal Ministry of Health (FMOH) is leading a sector-wide reform initiative aimed at dramatically enhancing the quality and availability of services at all levels of the country. One of the components of this guideline is to improve service quality, which is accomplished by conducting regular surveys on patients’ satisfaction [4]. Hospital pharmacies are an important part of the hospital complex system, which has a direct impact on patients’ satisfaction and the reputation of the hospital. Even though all professions exist to serve society’s needs, pharmacy professionals serving at hospital pharmacies can’t address the needs of society and individual patients. According to a study conducted at the Mizan-Tepi university teaching hospital in southern Ethiopia, nearly half of the clients who received pharmaceutical care services in the specified hospitals were not satisfied [5]. Another study found that the compassionate and respectful care of pharmacists provided to patients has an affirmative effect on client satisfaction while being a communicator and explaining was the second most important determinant [6]. The present study was commissioned by the hospital pharmacy department in order to improve its service to meet patient demand. The aim of this study was to assess patients’ satisfaction with pharmaceutical services and associated factors in public hospitals located in northwestern Ethiopia.

This will assist in bridging the gap between what patients require and what they receive. The findings are also useful in highlighting specific service difficulties that need to be addressed in order to provide high-quality pharmacy services.

## Methods and materials

### Study area

This study was carried out at the outpatient pharmacy of Debre Tabor comprehensive specialized hospital which is located 667 km northwest of Addis Ababa and 102 km from Bahir Dar (capital of Amhara regional state). It was founded by Italian missionaries in 1941 and serves a population of 2.3 million people in the zone and nearby districts. The hospital employed around 434 workers, with 296 of them being technical and 138 being supportive. There were 42 pharmacists among the technical staff. The hospital has one hundred twenty beds and six admission wards (medical, surgical, gynecology and obstetrics, pediatric, neonatal, and psychiatric) [7].

In this hospital, there are five pharmacies, namely, outpatient pharmacy, emergency pharmacy, inpatient pharmacy, antiretroviral treatment (ART) pharmacy, and gynecologic pharmacy, providing pharmaceutical services to patients. This study was carried out in the outpatient pharmacy of the hospital.

### Study design and period

An institution-based quantitative cross-sectional study was used. The study was carried out in outpatient pharmacy from January 1–June 30, 2021.

### Inclusion criteria

Patients above the age of 17 who were willing to engage in the study were included. Severely sick patients who were unable to participate in an interview and patients with mental disorders were excluded from the study. Patients who refused to give their consent were also excluded.

### Sample size determination and sampling procedure

Sample size (n) for the proportion of patients’ satisfaction was determined using the single population proportion formula considering the patients’ satisfaction of 51.9 percent (P) [5] at Mizan-Tepi university teaching hospital, 5 percent sampling error (d), and with a 95 percent confidence interval (Zα/2). The final sample size was 422, based on the assumption of a 10 percent nonresponse rate. As regards to the sample size for factors associated with patients’ satisfaction, it was computed using OpenEpi version 3 and considering 80 percent power of the study, 95 percent two-sided confidence interval, 1:1 case to control ratio, and 10 percent nonresponse rate. To determine sample size, the least odds ratio and proportion of cases with exposure to factors linked to patients’ satisfaction in prior studies were chosen. As a result, sample size was calculated by taking into account independent variables such as age, education, and occupation [8, 9]. Finally, the survey’s largest sample size (422) was considered. Study participants were selected by a systematic sampling technique; every fifth patient sequence was included in the study. The first patient was chosen by a lottery method.

### Data collection tool

Data was collected using a structured interview questionnaire, which was prepared after an intensive review of related literature on the topic [10, 11]. The tool contained two parts. The first part embraced questions about the socio-demographic profile of respondents, such as gender, age, place of residence, marital and educational status, and occupation. The second part was categorized into four subheadings. The first was to inquire about participants’ experiences with pharmacy services. The second was participants’ opinion towards the pharmacy setting, medication availability, and cost, whereas the third and fourth subheadings included questions to evaluate participants’ satisfaction towards the pharmacist approach or communication and medication instructions to evaluate the overall outpatient services provided to participants on their last visit, respectively. In all the satisfaction questions, the patients were asked to rate their satisfaction on a five-point Likert scale (1: very satisfied, 2: satisfied, 3: neutral, 4: dissatisfied, and 5: very dissatisfied). The five scales were combined into a three-scale structure for descriptive interpretation by combining "very satisfied" and "satisfied" as satisfaction, and "dissatisfied" and "very dissatisfied" as dissatisfaction. The data collection tool was initially prepared in an English version, which was later translated into an Amharic version and then retranslated to English to ensure consistency. The content validity was determined by a team of specialists from the fields of public health, pharmacy, epidemiology, and biostatistics. The final version of the questionnaire contained a reliable indicator, which was a good sign (as indicated by the Alpha Cronbach test value of 0.813).

### Data quality control and the data collection processes

Before the real data collection period, the questionnaire was pre-tested on 15 patients served in the outpatient pharmacy of Debre Tabor comprehensive specialized hospital to ensure its appropriateness. It was corrected and used after the pretest. The collected questionnaires were reviewed on a daily basis for completeness, accuracy, clarity, and consistency of data. Data was collected by two fourth-year Debre Tabor University undergraduate pharmacy students by face-to-face interview in the local language (Amharic) using a structured questionnaire guide.

### Data processing and analysis

The IBM SPSS statistical package (version 23) was used to enter and analyze the collected data [12]. The findings were presented using descriptive statistical methods (frequency, percentages, mean, mode, and median). The five-point scale satisfaction scores of each study participant were added and the mean of the sum was determined to find factors affecting satisfaction. The satisfaction score was then divided into two categories: contentment (less than or equal to 30) and dissatisfaction (greater than 30). The Shapiro-Wilk normality test revealed that our data were not normally distributed; thus, binary logistic regression was used to identify factors associated with satisfaction. The enter method was used. A reference variable was used to define the categorical variables, which was the last variable. An odds ratio and a p-value were used to test the association. In the univariate analysis, factors having a p-value < 0.2 were included in the multivariate analysis. The adjusted odds ratio and p < 0.05 were used to declare the final association.

### Ethical consideration

An ethical clearance letter (reference number: RCC821/21) was obtained from the research ethics review committee of the college of health sciences, Debre Tabor University. Because written consent is not required in such observational studies in our country, only informed verbal consent was obtained from the patients. The interview was not recorded on tape. Patients were informed that participation in the study was entirely voluntary and that they had the right to withdraw at any moment. Personal identities were not recorded on the questionnaire, and all data gathered through face-to-face interviews was kept private.

## Results

### Sociodemographic characteristics of participants

Out of 422 patients approached, 401 adult patients agreed to participate, giving a response rate of 95%. As can be seen from Table 1, more than half of the participants (n = 229, or 57.1%) were females. Among the respondents, those in the age group of 36 to 50 years old constituted the highest proportion (n = 143, 35.7%), followed by the age group of 26 to 35 years (n = 91, 22.7%). It is noticeable that the proportion of patients from urban areas was slightly higher than that from rural areas (n = 202, 50.4%), whereas one quarter of the participants were single (n = 106, 26.4%). As regards to the educational status of respondents, the most frequent had no formal education (n = 170, 42.4%), followed by those who had achieved certificates and above (n = 120, 29.9%).

**Table 1:** Sociodemographic characteristics of patients in Debre Tabor comprehensive specialized hospital in Northwest Ethiopia, 2021 (n = 401).

Variables	N (%)
Gender	
Male	172 (42.9)
Female	229 (57.1)
Age (years)	
18–25	85 (21.2)
26–35	91 (22.7)
36–50	143 (35.7)
Above 50	82 (20.4)
Place of residence	
Urban	202 (50.4)
Rural	199 (49.6)
Marital status	
Single	106 (26.4)
Married	215 (53.6)
Divorced	59 (14.8)
Widowed	21 (5.2)
Religion	
Orthodox	348 (86.8)
Muslim	35 (8.7)
Protestant	18 (4.5)
Educational status	
No formal education	170 (42.4)
Primary education	44 (11.0)
Secondary education	67 (16.7)
Certificate and above	120 (29.9)
Occupation	
No job	80 (20.0)
Government employee	82 (20.4)
Farmer	101 (25.2)
House wife	53 (13.2)
Merchant	61 (15.2)
Daily laborer	24 (6.0)

### Participants’ experiences with pharmacy services

Table 2 depicts the experience of patients during the service. Concerning their previous experiences with the outpatient pharmacy, a considerable portion of participants (n = 237, 59.1%) visited hospitals for chronic care. Regarding medication dispensed, (n = 195, 48.6%) patients received all the prescribed medications from the outpatient pharmacy units, whereas (n = 206, 51.4%) got some medication or none. Two hundred twenty-one (55.1%) of the participants were paid out-of-pocket for the medication dispensed. Finally, more than half of patients (n = 226, or 56.4%) spent more than 15 minutes at the outpatient pharmacy.

**Table 2:** Patient experiences with pharmacy services in Debre Tabor comprehensive specialized hospital in Northwest Ethiopia, 2021 (n = 401).

Variables	N (%)
Familiarity with institution	
First visit	164 (40.9)
Chronic care	237 (59.1)
Self-judged health status	
Severely sick	130 (32.4)
Sick	271 (67.6)
Medication dispensed	
All	195 (48.6)
None or some	206 (51.4)
Payment modality	
Out-of-pocket	221 (55.1)
Paid by insurance	144 (35.9)
Free	36 (9.0)
Waiting time	
<15min	175 (43.6)
>15min	226 (56.4)
Patients’ views on the requirement to improve the service*	
Improve medication availability	301 (75.1)
Increase waiting area space	284 (70.8)
Increase the number of staff	155 (38.6)
Reduce bureaucracy	137 (34.2)
Reduce waiting time	268 (66.8)

* The respondents can choose more than one option.

Patients were also asked regarding their view on what should be done to improve the quality of pharmacy services. As tabulated in Table 2, the need to improve medication availability was mentioned most frequently by nearly three-fourths of the participants (n = 301, 75.1%). The need to increase space in the waiting area, reduce waiting time, and increase the number of pharmacy staff were frequently referred to measures that patients thought would improve the quality of pharmacy service.

### Participants’ perception with pharmacy setting, drug availability, and cost

As it is indicated in Table 3, an uncomfortable and inconvenient private counseling area (n = 274, 68.3%) was the major complaint reported by study participants. On the other hand, more than half of the studied population agreed on the cleanness of the dispensary (n = 331, 82.5%), the pharmacy location convenience (n = 294, 73.3%), the adequate number of staff to the service (n = 236, 58.9%), and the comfortability and convenience of the waiting area (n = 221, 55.1%). Among the 401 study participants, 221 got their medication by paying in cash. Among them, (n = 110, 49.8%) replied that the cost of the medication was affordable. Furthermore, nearly one-fourth of the participants believed that the number of pharmacy staff was not adequate to provide the services.

**Table 3:** Study participants’ opinions towards the pharmacy setting, medication availability, and cost, 2021 (n = 401).

Variables (n = 401)	Yes	Somewhat/Neutral	Not
The pharmacy location is convenient	294 (73.3)	17 (4.3)	90 (22.4)
The private counseling area is comfortable and convenient	106 (26.4)	21 (5.3)	274 (68.3)
The waiting area is comfortable and convenient	221 (55.1)	22 (5.5)	158 (39.4)
The pharmacy is clean	331 (82.5)	38 (9.5)	32 (8.0)
Medications I need are available	138 (34.4)	164 (40.9)	99 (24.7)
The cost of the medication is fair (n = 221)	110 (49.8)	38 (17.2)	73 (33.0)
The staff numbers are enough to provide the service	236 (58.9)	57 (14.2)	108 (26.9)

### Satisfaction scores of participants toward the pharmacist’s approach

As illustrated in Table 4, the vast majority of the study participants had a high satisfaction on the voice tone (n = 377, 94.0%), availability on the workplace of the pharmacists (n = 361, 90.0%), politeness (n = 334, 83.3%), and equitable service delivery (n = 324, 80.8%). They also had a high satisfaction on the patient handling (n = 321, 80.0%).

**Table 4:** Study participants’ satisfaction towards the pharmacist’s approach or communication.

Variables	Satisfied	Neutral	Dissatisfied
The politeness and interest of the pharmacist were good	334 (83.3)	10 (2.5)	57 (14.2)
Pharmacists provide service equally	324 (80.8)	14 (3.5)	63 (15.7)
Pharmacists treat the patient with dignity and respect	321 (80.0)	14 (3.5)	66 (16.5)
Pharmacy professionals were available during the visit	361 (90.0)	20 (5.0)	20 (5.0)
The voice and tone of the pharmacy personnel were clear	377 (94.0)	4 (1.0)	20 (5.0)
The wait time in the pharmacy was fair	197 (49.1)	14 (3.5)	190 (47.4)

As illustrated in Table 4, the vast majority of the study participants had high satisfaction with the voice tone (n = 377, 94.0%), availability of the pharmacists’ workplace (n = 361, 90.0%), politeness (n = 334, 83.3%), and equitable service delivery (n = 324, 80.8%). They also had high satisfaction with the patient handling (n = 321, 80.0%).

### Satisfaction of participants toward pharmacists’ medication advice

Although the study participants had high satisfaction (n = 328, 81.8%) towards the language of communication, the emphasis given on the medications they received (n = 294, 73.3%), and the labeling instructions (n = 249. 62.1%), they had a lower level of satisfaction towards most of the guidance they received about their medications. Less than half of the study participants with the time given (n = 165, 41.1%) and the medication storage conditions (n = 130, 32.4%) were satisfied. Furthermore, only about a quarter of those who took part in the study were satisfied with the pharmaceutical precautions and side effects (n = 97, 24.2%), and drug-drug and drug food interactions (n = 94, 23.4%) (Table 5).

**Table 5:** Study participants’ satisfaction with the pharmacist’s medication instructions.

Variables	Satisfied	Neutral	Dissatisfied
Counseling time was enough	165 (41.1)	77 (19.2)	159 (39.7)
The pharmacist ensures that medications are taken as prescribed	294 (73.3)	48 (12.0)	59 (14.7)
The pharmacist told me about proper storage of medications	130 (32.4)	47 (11.7)	224 (55.9)
The pharmacist tells about medication precautions and side effects	97 (24.2)	48 (12.0)	256 (63.8)
Drug-drug and drug-food interactions	94 (23.4)	54 (13.5)	253 (63.1)
Instructions that are readable and understandable on the label	249 (62.1)	19 (4.7)	133 (33.2)
Give administration instructions in an understandable language	328 (81.8)	49 (12.2)	24 (6.0)

### The sum of patient satisfaction ratings for outpatient pharmacy services

Based on the five-point Likert scale, the overall mean score of satisfaction was 30.6 out of a maximum of 100 scores. By taking this mean score as a cut-off point, 204 (50.9%) of the study participants had satisfaction with the outpatient pharmacists’ service. The remaining patients (n = 197, 49.1%) were found to be dissatisfied with the services, scoring above the mean with a standard deviation of 8.7 at 95% CI (24.2, 33.8) (Table 6).

**Table 6:** Study participants’ satisfaction scores towards pharmacist services.

The summed satisfaction	Frequency (N = 401)	Percent
Mean score (Std. Deviation)	30.6±8.7	
Median score (Range)	30.00 (21–56)	
Mode	28.00	
Satisfaction score ≤ 30 (Satisfied)	204	50.9
Satisfaction score > 30 (Dissatisfied)	197	49.1

### Factors affecting participants’ satisfaction

Variables with a p-value less than 0.2 in the univariate model were included in the final multivariate model as shown in Table 7. As a result, all the socio-demographic variables, variables toward the pharmacist’s approach, and pharmacist medication advice were excluded from the model. Participants’ responses scored on the uncomfortable and inconvenient waiting areas [AOR = 0.31; 95%CI, 0.13, 0.49)] were found to be negatively associated with the level of patients’ satisfaction. Also, participants’ responses to the medication availability were scored as somewhat fair [AOR = 0.51; 95%CI, (0.16, 1.97)], and the unavailability of medications [AOR = 0.12; 95%CI, (0.02, 0.37)] was negatively associated with the respondent satisfaction. Uncomfortable and inconvenient private counseling areas [AOR = 1.37; 95%CI, (0.79, 4.42)] showed a negative association with their satisfaction.

**Table 7:** Association test of study participants’ satisfaction with pharmacist services in Debre Tabor comprehensive specialized hospital in Northwest Ethiopia, 2021 (n = 401).

Factors/variables	Frequency (%)		COR (95% CI)	P-value	AOR (95% CI)	P-value
	Satisfied	Dissatisfied				
Medications I need are available						
Not	30 (14.7)	68 (34.5)	0.22 (0.06-0.87)	0.020	0.12 (0.02-0.37)	0.040
Somewhat/Neutral	94 (46.1)	71 (36.1)	0.41 (0.26, 0.87)	0.002	0.51 (0.16-1.97)	0.001
Yes	80 (39.2)	58 (29.4)	1.00	0.010	1.00	0.020
The staff numbers are enough to provide the service						
Not	34 (16.6)	68 (34.5)	0.55 (0.32, 0.91)	0.021	0.66 (0.37, 1.25)	0.521
Somewhat/Neutral	24 (11.8)	39 (19.8)	1.51 (1.01,2.32)	0.035	0.64 (0.04, 1.09)	0.125
Yes	146 (71.6)	90 (45.7)	1.00	0.821	1.00	0.575
The pharmacy location is convenient						
Not	14 (6.9)	76 (38.6)	1.06 (0.65, 1.72)	0.421	1.28 (0.24, 6.18)	0.372
Somewhat/Neutral	7 (3.4)	11 (5.6)	1.47 (0.91, 2.27)	0.835	1.61 (0.29, 3.42)	0.621
Yes	183 (89.7)	110 (55.8)	1.00	0.125	1.00	0.273
The private counseling area is comfortable and convenient						
Not	103 (50.5)	170 (86.3)	1.05 (0.25, 4.34)	0.061	1.37 (0.79-4.42)	0.030
Somewhat/Neutral	12 (5.9)	9 (4.6)	0.56 (0.16-1.93)	0.452	0.51 (0.32, 0.97)	0.142
Yes	89 (43.6)	18 (9.1)	1.00	0.063	1.00	0.213
The waiting area is comfortable and convenient						
Not	34 (16.7)	125 (63.4)	0.35 (0.78-0.99)	0.010	0.31 (0.13, 0.49)	0.010
Somewhat/Neutral	12 (5.9)	10 (5.1)	0.71 (0.42, 1.12)	0.002	0.67 (0.41, 2.02)	0.002
Yes	158 (77.4)	62 (31.5)	1.00	0.241	1.00	0.213
The pharmacy is clean						
Not	3 (1.5)	29 (14.7)	0.45 (0.28, 0.71)	0.014	0.53 (0.30, 1.17)	0.213
Somewhat/Neutral	5 (2.4)	33 (16.8)	0.49 (0.24, 0.98)	0.012	0.52 (0.27, 1.19)	0.061
Yes	196 (96.1)	135 (68.5)	1.00	0.015	1.00	0.320
The cost of the medication is fair						
Not	17 (9.0)	56 (26.3)	0.83 (0.94-2.39)	0.132	1.49 (0.69, 3.24)	0.155
Somewhat/Neutral^†^	106 (56.4)	112 (52.6)	0.56 (0.16-1.92)	0.297	0.82 (0.43-1.57)	0.235
Yes	65 (34.6)	45 (21.1)	1.00	0.041	1.00	0.673

COR = crude odds ratio, AOR = adjusted odds ratio, CI = confidence interval,

^†^Those respondents taking the medications for free were classified as neutral.

## Discussion

Assessing patients’ satisfaction with pharmacy services is essential to improving the quality of current services. This will help to bridge the gap between what patients require and what they actually receive [13].

This study was a cross-sectional study designed to assess patients’ satisfaction toward outpatient pharmacy services to obtain an insight into the quality of the healthcare service provided at Debre Tabor comprehensive specialized hospital outpatient pharmacy. Two parameters of questions were used to assess the patients’ satisfaction. The first was about the pharmacists’ approach or communication, and the second was on the medication guidance given. The satisfaction level (50.9%) of patients in this study was almost consistent with the finding from a study done in Tikur Anbessa specialized hospital, which reported a satisfaction level of 51.6% [9]. The current level of satisfaction was slightly greater than the survey conducted at Yekatit 12 referral hospital (47%) [14], slightly lower than the study in Mizan-Tepi university teaching hospital (52.6%) [7] and Wolaita Sodo university teaching hospital (54.2%) [15]. On the other hand overall result of patients’ satisfaction regarding pharmacy services was lower than different study finding in Ethiopia [16, 17]. The discrepancy in satisfaction levels seen could be due to the fact that the data collection instrument utilized in each study differs, which could have influenced the results. In contrast to this study’s findings and the above-cited references in the country, a nationwide survey found that 74.5% of patients were satisfied [18]. The parameters used to assess satisfaction are likely to be the source of this disparity. The dispensing space, dispensing process, privacy of the setting, personnel skills, and support supplied to the patient were the factors utilized to determine patients’ satisfaction in the countrywide survey. All of the above factors differ from one institution to another. When compared to findings in Brazil (58.4%), South Korea (74.6%), Spain (76%), and the United Arab Emirates (77.1%), the current finding is the lowest [19–22]. The higher level of satisfaction in these studies could indicate that developed countries have better pharmacy services than developing countries like Ethiopia [23].

Although satisfaction with the pharmacist’s approach or communication was consistent and rated higher in the present study, mixed and extremely low ratings were noticed for the medication guidance offered to them. This was a warning message that may have made the whole pharmacy service poor and valueless. Despite the fact that nearly half of the respondents (49.1%) were dissatisfied with the overall pharmacy service, the patients’ satisfaction with some of the most important medication instructions, such as administration instructions (81.8%), the provision of instructions on how to take medications (73.3%), and label clarity (62.1%), was higher. In support of this finding, a nationwide study on the quality of pharmaceutical services found that patients had a higher knowledge score for the dose (83.2%), frequency (90.8%), and route of administration (94.8%) [18]. The study conducted in Tikur Anbessa specialized hospital also showed 83.2% and 67.6% of patients were satisfied with the administration instructions and label clarity, respectively [9]. A study conducted in Malta also found that 94% of patients were satisfied with the informational instruction on how to take medication [24].

Around three-quarters of the respondents are satisfied with the pharmacist’s treatment of them with decency and respect. Closer to our findings, a study in Gondar found that 66.2% of clients were satisfied with the respectful manner of the pharmacy staff [11]. A survey conducted in Malaysia found that 91.5% of patients were satisfied with the professional manner of pharmacists [25]. In contrast, less than half of the patients in the present study were satisfied with the counseling time (n = 165, 41.1%) and the proper medication storage conditions (n = 130, 32.4%). Furthermore, only about a quarter of the patients in the current study were satisfied with the medication precautions and side effects (n = 97, 24.2%) and drug-drug interactions and food not to be taken with drugs (n = 94, 23.4%). Similarly, a published study finding conducted in Gondar showed that 62% of respondents were dissatisfied with the information given by the pharmacist about the possible adverse effects of medications [11]. The lower satisfaction rate in these regards in our setting might have resulted from the absence of a separate counseling area in the outpatient pharmacy of Debre Tabor comprehensive specialized hospital, which may have hindered the pharmacists’ ability to have detailed communication with the patient. In line with these findings, the nationwide and Tikur Anbessa specialized hospital studies found that the majority of patients interviewed did not know the names, precautions, and storage conditions of the medications that were dispensed to them [9, 18]. This suggests that medication guidance is one area that needs to be improved in order to increase overall patients’ satisfaction. Other studies in Addis Ababa and Gondar also found a need for improvement in the medication guidance area [9, 26]. The satisfaction of study participants was also investigated for relevant determinants. Unlike a number of previous studies [5, 14, 16, 17, 26], no significant association was seen between socio-demographic variables and perceived level of satisfaction in this study. Similarly, research conducted in Gondar and Tikur Anbessa specialized hospitals indicated 51.9% and 51.6% of patients were adequately satisfied with general services, irrespective of age and gender, respectively [9, 11]. According to the studies done in South Korea and Malaysia [19, 25], there was no significant association between respondents’ level of satisfaction and their age, gender, and educational status.

On the system-related aspects, on the other hand, study participants’ belief in drug availability, staff number sufficiency, cleanness of the pharmacy, and cost of medication were found to be associated with satisfaction in the univariate model. In line with this, a study conducted in Yekatit 12 hospital revealed that drug availability shows a statistically significant association with patients’ satisfaction in the multivariate model [AOR 1.9; 95% CI, (1.12–2.88)] [14]. Similarly, various studies conducted in Ethiopia at various times discovered a significant association between medication availability and the overall level of satisfaction [11, 14, 16, 27, 28]. This is in contrast to the findings of the studies conducted in South Korea and Nigeria, which revealed that there was no significant association between drug availability and patients’ satisfaction [19, 29]. Variation in this finding could be attributable to the presence of an equipped pharmacy service (supply and medicine) as well as patient demography variation. As a result, the current finding implies that drug availability is a key service with which patients are more satisfied.

Furthermore, in both univariate and multivariate model analysis, only study participant belief in waiting area conformability was significantly associated with satisfaction score in the current study. The significant association of patients’ dissatisfaction with the uncomfortable waiting area may be explained in agreement with the nationwide and Tikur Anbessa specialized hospital studies [9, 18]. This means that the dispensary area needs to be improved to meet the needs of the patients.

### Limitations of the study

The limitations of this study are recognized by the authors. First and foremost, this single-centered study was conducted in one setting, and the results will not be generalized to other settings. Other factors that could influence satisfaction, such as a pharmacist’s qualification, years of experience, and training experience, were not addressed. Furthermore, because the interview was conducted on hospital premises, the results of this study may be prone to social desirability bias.

## Conclusion

In this study, the patients’ satisfaction levels with pharmacy service were found to be greater than 50%. Although this figure was comparable to other studies in the country, it was much lower than the nationwide study and the average standard level expected. Respondents’ satisfaction with the pharmacist’s approach or communication skills was highly encouraging. However, this was compromised by the lower satisfaction report towards the medication guidance given. The socio-demographic characteristics of patients have no association with their level of satisfaction, but their perception of uncomfortable private counseling areas and waiting areas was negatively associated with their satisfaction. It is recommended that hospital management ensure the outpatient pharmacy setting conformability and the delivery of proper and sufficient medication guidance to needy patients. Moreover, medication availability was an independent variable strongly linked to patients’ satisfaction. The hospital administration should institute a better service provision system in relation to medication accessibility and availability.

## Supporting information

S1 File Data collection tool (questionnaire). (DOCX)
