# Supplementary material for: Patients’ satisfaction with outpatient pharmacy services and associated factors in Debre Tabor comprehensive specialized hospital, Northwest Ethiopia: A cross-sectional study
Source: PLoS One. 2022 Jan 5;17(1):e0262300. doi: 10.1371/journal.pone.0262300 (PMC8730437; doi:10.1371/journal.pone.0262300)
Supplement: S1 File — (DOCX) [file pone.0262300.s001.docx]

**Patients’ satisfaction with outpatient pharmacy services and associated factors in Debre Tabor comprehensive specialized hospital, Northwest Ethiopia: A cross-sectional study**

**Sociodemographic characteristics of patients in Debre Tabor comprehensive specialized hospital in Northwest Ethiopia.**

1. Gender
2. Male
3. Female
4. Age (years)
5. 18–25
6. 26–35
7. 35–50
8. Above 50
9. Place of residence
10. Urban
11. Rural
12. Marital status
13. Single
14. Married
15. Divorced
16. Widowed
17. Religion
18. Orthodox
19. Muslim
20. Protestant
21. Educational status
22. No formal education
23. Primary education
24. Secondary education
25. Certificate and above
26. Occupation
27. No job
28. Government employee
29. Farmer
30. House wife
31. Merchant
32. Daily laborer

**Patient experiences with pharmacy services in Debre Tabor comprehensive specialized hospital in Northwest Ethiopia.**

1. Familiarity with institution
2. First visit
3. Chronic care
4. Self-judged health status
5. Severely sick
6. Sick
7. Medication dispensed
8. All
9. None or some
10. Payment modality
11. Out-of-pocket
12. Paid by insurance
13. Free
14. Waiting time
15. <15min
16. >15min
17. Patients’ views on the requirement to improve the service*
18. Improve medication availability
19. Increase waiting area space
20. Increase the number of staff
21. Reduce bureaucracy
22. Reduce waiting time

* The respondents can choose more than one option.

**Study participants’ opinions towards the pharmacy setting, medication availability, and cost.**

| S.No. | Questions | 1 | 2 | 3 | 4 | 5 |
| --- | --- | --- | --- | --- | --- | --- |
|  | The pharmacy location is convenient |  |  |  |  |  |
|  | The private counseling area is comfortable and convenient |  |  |  |  |  |
|  | The waiting area is comfortable and convenient |  |  |  |  |  |
|  | The pharmacy is clean |  |  |  |  |  |
|  | Medications I need are available |  |  |  |  |  |
|  | The cost of the medication is fair |  |  |  |  |  |
|  | The staff numbers are enough to provide the service |  |  |  |  |  |

Key: 1: very satisfied, 2: satisfied, 3: neutral, 4: dissatisfied, and 5: very dissatisfied

**Study participants’ satisfaction towards the pharmacist’s approach or communication.**

| S.No. | Questions | 1 | 2 | 3 | 4 | 5 |
| --- | --- | --- | --- | --- | --- | --- |
|  | The politeness and interest of the pharmacist were good |  |  |  |  |  |
|  | Pharmacists provide service equally |  |  |  |  |  |
|  | Pharmacists treat the patient with dignity and respect |  |  |  |  |  |
|  | Pharmacy professionals were available during the visit |  |  |  |  |  |
|  | The voice and tone of the pharmacy personnel were clear |  |  |  |  |  |
|  | The wait time in the pharmacy was fair |  |  |  |  |  |

Key: 1: very satisfied, 2: satisfied, 3: neutral, 4: dissatisfied, and 5: very dissatisfied

**Study participants’ satisfaction with the pharmacist’s medication instructions.**

| S.No. | Questions | 1 | 2 | 3 | 4 | 5 |
| --- | --- | --- | --- | --- | --- | --- |
|  | Counseling time was enough |  |  |  |  |  |
|  | The pharmacist ensures that medications are taken as prescribed |  |  |  |  |  |
|  | The pharmacist told me about proper storage of medications |  |  |  |  |  |
|  | The pharmacist tells about medication precautions and side effects |  |  |  |  |  |
|  | Drug-drug and drug-food interactions |  |  |  |  |  |
|  | Instructions that are readable and understandable on the label |  |  |  |  |  |
|  | Give administration instructions in an understandable language |  |  |  |  |  |

Key: 1: very satisfied, 2: satisfied, 3: neutral, 4: dissatisfied, and 5: very dissatisfied
